# Mass Spectrometry Reveals Molecular Structure of Polyhydroxyalkanoates Attained by Bioconversion of Oxidized Polypropylene Waste Fragments

**DOI:** 10.3390/polym11101580

**Published:** 2019-09-27

**Authors:** Brian Johnston, Iza Radecka, Emo Chiellini, David Barsi, Vassilka Ivanova Ilieva, Wanda Sikorska, Marta Musioł, Magdalena Zięba, Paweł Chaber, Adam A. Marek, Barbara Mendrek, Anabel Itohowo Ekere, Grazyna Adamus, Marek Kowalczuk

**Affiliations:** 1 Wolverhampton School of Biology, Chemistry and Forensic Science, Faculty of Science and Engineering, University of Wolverhampton, Wolverhampton WV1 1LY, UK; B.Johnston@wlv.ac.uk (B.J.); Barbara.Mendrek@wlv.ac.uk (B.M.); I.A.Jonah@wlv.ac.uk (A.I.E.); 2 Laboratorio Materiali Polimerici Ecocompatibili (LMPE), via Nuova, 44/a, Segromigno in Monte, 55018 Capannori (LU), Italy; emo.chiellini@lmpe.eu (E.C.); david.barsi@lmpe.eu (D.B.); Vassilka.Ilieva@lmpe.eu (V.I.I.); 3 Centre of Polymer and Carbon Materials, Polish Academy of Sciences, 41-800 Zabrze, Poland; wsikorska@cmpw-pan.edu.pl (W.S.); m.musiol@cmpw-pan.edu.pl (M.M.); mzieba@cmpw-pan.edu.pl (M.Z.); pchaber@cmpw-pan.edu.pl (P.C.); 4 Department of Chemical Organic Technology and Petrochemistry, Silesian University of Technology, 44-100 Gliwice, Poland; adam.a.marek@polsl.pl

**Keywords:** electrospray ionization dual mass spectrometry (ESI-MS/MS), polyhydroxyalkanoate (PHA), polypropylene (PP), prodegraded, *Cupriavidus necator*, coral reef, bioplastics, recycling

## Abstract

This study investigated the molecular structure of the polyhydroxyalkanoate (PHA) produced via a microbiological shake flask experiment utilizing oxidized polypropylene (PP) waste as an additional carbon source. The bacterial strain *Cupriavidus necator* H16 was selected as it is non-pathogenic, genetically stable, robust, and one of the best known producers of PHA. Making use of PHA oligomers, formed by controlled moderate-temperature degradation induced by carboxylate moieties, by examination of both the parent and fragmentation ions, the ESI-MS/MS analysis revealed the 3-hydroxybutyrate and randomly distributed 3-hydroxyvalerate as well as 3-hydroxyhexanoate repeat units. Thus, the bioconversion of PP solid waste to a value-added product such as PHA tert-polymer was demonstrated.

## 1. Introduction

Mass spectrometry (MS) currently represents a powerful technique for the structural characterization of polymers. Progress in this area is tremendous and covers novel mass spectrometry characterization methods as well as the polymers that have already been analyzed. With a soft ionization technique such as electrospray ionization (ESI), and a reasonable mass accuracy (<100 ppm), single-stage MS can provide information about (co)polymer structures [1]. It may also be helpful in the synthesis of biodegradable polymers from natural and nonrenewable resources [2].

Dual-stage electrospray mass spectrometry (ESI-MS/MS), which exploits the dissociation chemistry of mass-selected ions, has been effectively used to analyze the structures of polymer end groups and the sequences of linear copolymers [3]. It is of particular importance in the case of biodegradable polyesters and the polymeric materials made from them due to their potential applications in medicine, the cosmetics industry, agro chemistry, packaging, and environmental protection. In MS/MS studies, mass analysis is performed twice. The parent ion formed in the ion source is mass-selected and its internal energy is raised to induce fragmentation. Among common ion-activation methods, collisionally activated dissociation (CAD) has been preferentially used in our studies [4]. By means of the methodology described by some of us, the random comonomer arrangements have been confirmed in poly(3-hydroxybutyrate-co-3-hydroxyvalerate), (PHBV), and poly(3-hydroxybutyrate-co-3-hydroxyhexanoate), (PHBH), copolymers (contained by two different co-monomeric units) and was also applied to differentiate block from random copolymers [5,6]. However, to the best of our knowledge, deeper MS studies of PHA with more than two different repeat units have not been performed until now.

**Figure 1:**
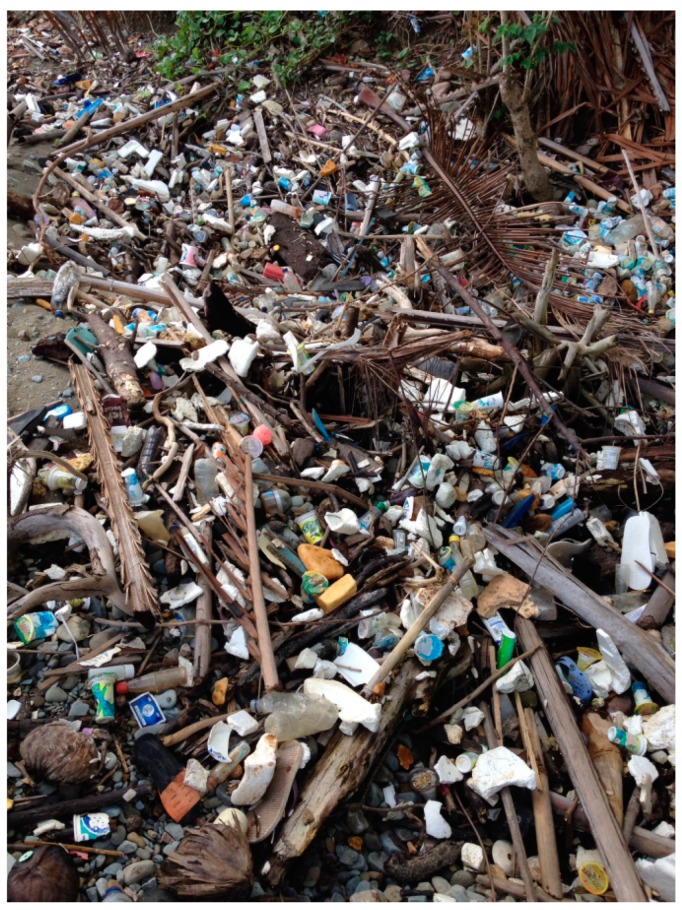
Beach area in Ambon, Indonesia contaminated with plastic waste. Image provided by Chris Mason-Parker.

Polypropylene (PP) is the second-most widely manufactured petroleum-based plastic after polyethylene (PE) and some of its applications include polymer banknotes, clothing, bottles, packaging, carpets, and automotive products [7,8]. As with many other traditionally produced plastics, PP is cost-effective, lightweight, and strong, however, its highly stable structure makes the process of natural biological degradation difficult [9]. As a result of this, PP production creates a large carbon footprint and due to the short lifespan of packaging, the majority of PP ends up in landfills [8,10]. Approximately 8 million tons of mixed plastic ends up in our oceans and coastal areas (Figure 1), and the majority of the floating debris is composed of PP and PE due to their high production volumes and buoyancy [10,11]. During a recent survey of 159 coral reefs in the Asia–Pacific region, it has been estimated that there are approximately 11.1 billion plastic items entangled in the coral, and this figure is expected to rise by 40 percent by 2025 [12]. This plastic waste does not just pose a threat due to its presence by choking or poisoning sea-life, but it can also host pathogens that are now known to be initiators of disease outbreaks across coral reefs [12,13]. PP has even been directly identified as a transporter of pathogenic bacteria related to cholera responsible for white syndromes, diseases that destroy coral tissue [12,13,14,15]. The potential use of PP in this study is for its bioconversion into value-added products such as polyhydroxyalkanoates (PHAs), which would leave vital food-related carbon resources untouched.

PHAs are a family of organic polymers that contain 3-, 4-, 5-, and 6-hydroxyalkanoic acids. They are non-toxic, biocompatible, and most importantly, completely biodegradable, making them a “greener” substitute for common fossil-fuel-based synthetic plastics. PHAs are produced by bacteria and plants naturally from renewable resources [16]. The bacterial strain *Cupriavidus necator* H16 (formerly *Ralstonia eutropha*) was selected for this study as it is a reliable synthesizer of PHAs (used as an energy store), it grows well at relatively low temperatures, has some immunity to some heavy metals, and possesses a stable, well-documented genetic profile [17,18]. In order to demonstrate PHA production, shake flask experiments were used as they are easily repeatable and provide partial stress conditions through a limited oxygen supply. This limitation can be advantageous as it is already known that some bacteria can accumulate large amounts of PHA under oxygen limitation conditions. A disadvantage of using shake flasks is that conditions monitoring is limited, however, for the purposes of this study, the required parameters were maintained. The oxidatively prodegraded PP fermentation, herein described, could be one way according to which plastic solid waste (PSW) could be recycled for value-added PHA productions [19,20,21].

While traditional techniques such as gel permeation chromatography (GPC) and nuclear magnetic resonance (NMR) spectroscopy are powerful tools for the structural characterization of PHAs (average across a whole sample), mass spectrometry offers the ability to make absolute mass measurements at a molecular level. Since each co-monomer exhibits different dissociation behavior, fragmentation pathways along the chain usually allow copolymers to be sequenced and dual-stage mass spectrometry (MS/MS) can provide detailed structural information about the repeat unit chemistry, end groups, and backbone topology [22,23,24].

The ESI-MS/MS structural studies of bacterial PHA copolymers were based on the analysis of their oligomers obtained by alkaline hydrolysis or controlled moderate-temperature degradation induced by carboxylate moieties [25]. Using this approach, oligomers containing carboxylic and olefinic end groups and the same composition and sequence distribution as the starting materials were obtained as revealed by ^1^H-NMR and ESI-MS/MS analysis [3]. This relatively rapid approach, developed at our laboratories, has been recently adopted for the automated routine analysis of PHBV copolymers by other authors [26]. In this study, the ESI-MS/MS data acquired enabled the corroboration of whether the obtained PHA copolymers consisted of two (HB and HV) or three (HB, HV, and HH) co-monomer units randomly distributed or assembled in a block type structure.

## 2. Materials and Methods

### 2.1. PP Carbon Source

**Figure 2:**
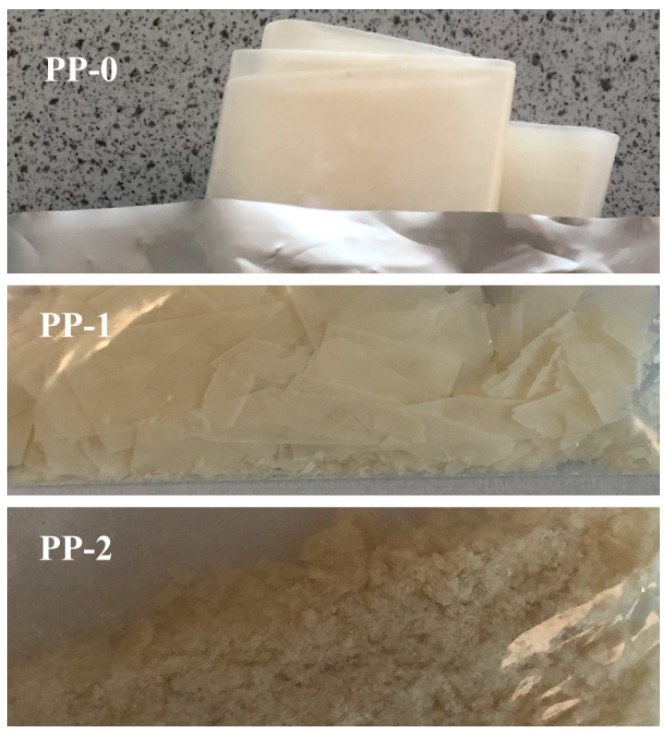
Images of the polypropylene (PP) samples used as additional carbon sources for polyhydroxyalkanoate (PHA) production. PP-0 was the original sample received and PP-1 and PP-2 were treated thermally.

The original oxidatively prodegraded PP (PP-0) sample was kindly supplied by the Laboratorio Materiali Polimerici Ecocompatibili (LMPE, Capannori, Italy). The PP film samples contained 1% by weight of cobalt stearate as pro-oxidant/pro-degradant additive. The sample had a melt flow index (MFI) of 35 g/10 min at 180 °C and a density of 0.900 g/cm^3^. PP-0 was then subjected to thermal oxidation and the successful prodegraded samples were designated PP-1 and PP-2, as shown in Figure 2.

The further oxidation treatment was conducted at the Department of Chemical Organic Technology and Petrochemistry, Silesian University of Technology Gliwice, Poland. The PP-0 films (10 g) were placed in a 100 mL glass-reactor and immersed in a thermal bath (60 and 80 °C). The process was carried out in a two-phase system: gas-solid phase. Oxygen–ozone mixture (75 mgO_3_/L) was introduced into the reactor at a flow rate of 7.5 L/h and the overall duration of the processes was 20 h. The basic property of the oxidized products was the acid number (AN), which was determined according to PN-EN ISO 2114:2002 standard “Plastics (polyester resins) and paints and varnishes (binders)–Determination of partial acid value and total acid value”. The PP-0 to 2 structural details can be found in Section 3.1, Section 3.2 and Section 3.3.

### 2.2. Selected Microorganism

The species of bacteria used was *C. necator* H16 (NCIMB 10442, ATCC 17699). This organism was obtained from the University of Wolverhampton culture storage facility where it was freeze-dried at −20 °C. Cultures were revived and grown at 30 °C in nitrogen-rich tryptone soya broth (TSB) media at 150 rpm. The strain was later sub-cultured onto tryptone soya agar (TSA) in Petri dishes and incubated at the standard optimal temperature for *C. necator* (30 °C) for 24 h. At this point, the strain was ready for use to inoculate microbial experiments in TSB and nitrogen-limited, basal salts media (BSM) during fermentations of 48 h.

### 2.3. Growth Media and Chemicals

The TSB and TSA for bacterial growth were purchased from Lab M Ltd. (Lancashire, UK). Both of these growth media were prepared using aseptic techniques under standard conditions. The BSM used consisted of distilled water, 1 g/L K_2_HPO_4_, 1 g/L KH_2_PO_4_, 1 g/L KNO_3_, 1 g/L (NH_4_)_2_SO_4_, 0.1 g/L MgSO_4_7H_2_O, 0.1 g/L NaCl, and 10 mL/L of a trace element solution that contained 2 mg/L CaCl_2_, 2 mg/L CuSO_4_5H_2_O, 2 mg/L *Mn*SO_4_5H_2_O, 2 mg/L ZnSO_4_5H_2_O, 2 mg/L FeSO_4_, and 2 mg/L (NH_4_)_6_Mo_7_O_24_ 4H_2_O. The BSM salts used were obtained from BDH Chemicals Ltd. (Poole, UK). The Ringer’s solution used throughout the study was purchased from Lab M Ltd. (Lancashire, UK). A 1/4 strength tablet was added to 500 mL of deionized water and dissolved for regular colony counting (Section 2.4). All of the media mentioned were autoclaved (Priorclave Ltd., London, UK) at 121 °C for 15 min. From 25 mL starter flasks of TSB grown at 30 °C, 1 mL of inocula were dropped into 250 mL TSB media conical flasks to establish a narrow starting range of CFUs for comparison. 

### 2.4. Shake Flask Procedure

Starter cultures were prepared using 20 mL TSB (in 50 mL flasks). These flasks were inoculated with single *C. necator* colonies from TSA spread plates. Those cultures were then incubated (aerobically) for 24 h at 30 °C at 150 rpm, in a rotary incubator (Incu-Shake MIDI, Shropshire, UK). After 24 h, these cultures were checked for contamination by standard Gram staining and microscope techniques. Shake flask experimentation was performed in triplicate using 500 mL wide neck Erlenmeyer flasks. Each flask had 1 g of the appropriate PP sample, that was first put into a 100 mL beaker and sonicated (in 50 mL of the appropriate media) for 8 min at 0.5 active and passive intervals with a power of 70% using a Bandelin Electronic sonicator (Berlin, Germany) to form an emulsion of either TSB/PP-0 to 2 or BSM/PP-0 to 2. This emulsion was then sampled for sterility by spread plating. The sterile emulsion was then added to 200 mL of sterile TSB or BSM in a 500 mL flask, followed by 1000 µL of the starter culture, giving a total volume of 250 mL and an equal concentration of bacteria for comparison. The experimental control was 200 mL TSB or BSM, inoculated with 1000 µL of starter culture with no PP samples. The flasks were incubated in a rotary incubator under the standard growth conditions for *C. necator* for 48 h.

The viable cell counts were undertaken using the techniques of Miles and Misra [27]. In summary, 5 mL samples were aseptically collected from flask cultures at 0, 3, 24, 27, and 48 h. Each sample was serially diluted from 10^−1^ to 10^−9^ and then 20 µL of each dilution was pipetted onto sectioned TSA plates in triplicate. These plates were then incubated at 30 °C for 48 h and the colonies were counted and expressed in log10 CFU mL^−1^ (Section 3.4).

### 2.5. PHA Extraction Method

A 48 h shake flask period was found to give optimum PHA yield. Flasks were removed from their incubators and their contents were filtered with a sieve to remove PP residue material. The filtrate was then divided into tubes and centrifuged in a Sigma 6-16KS centrifuge for 10 min at room temperature at 4500 rpm. The supernatant was discarded and the biomass pellet was frozen overnight at −20 °C. The biomass was then lyophilized in an Edwards freeze-drier (Modulyo, Crawley, UK) for a 48 h period at −40 °C and 5 mbar. The dried-out biomass was then carefully weighed and recorded as cell dry weight (CDW). For extraction, the sample was placed inside a thimble closed with cotton (to serve as a filter) and using Soxhlet extraction, HPLC grade chloroform was heated to 47–50 °C for 48 h. The PHA removed from the biomass was collected as a chloroform/biopolymer mixture. Rotation evaporation (at 50 °C) was used to remove any chloroform from the round bottom flask. Biopolymer precipitation using *n*-hexane was used in a 250 mL round-bottom flask and then this was separated with filtration (using Whatman No. 1 paper, Whatman Laboratory, Maidstone, Kent, UK). The final PHA sample was then left in a sterile fume cupboard for drying and the yield was recorded using the following formula:

Percentage yield = [(weight of extracted polymer)/(dry cell biomass weight)] × 100

### 2.6. Characterization Methods

#### 2.6.1. GPC Analysis

The molar mass and molar mass distribution of the PP samples were determined by gel permeation chromatography (GPC, Agilent Technologies PL GPC 220) apparatus equipped with two columns: Agilent PLgel Olexis guard plus 3x Olexis, 30 cm, 13 µm, at 160 °C. 1,2,4-Trichlorobenzene with anti-oxidant was employed as a solvent at a flow rate of 1.0 mL/min. The data were analyzed using polystyrene calibration.

The GPC experiments for the PHA-PP samples studied were conducted in a chloroform solution at 35 °C at a flow rate of 1 mL/min using a Viscotek VE 1122 solvent delivery system with two Mixed C PL-gel styragel columns in series and a Shodex SE 61 refractive index detector. A volume of 10 μL of sample solutions in CHCl_3_ (concentration 0.5% *m/v*) was injected into the system. Polystyrene standards with low dispersity were used to generate a calibration curve.

#### 2.6.2. FTIR Analysis

Fourier-transform infrared spectroscopy (FTIR) was used to record the absorbance spectra at an accumulation setting of 64 scans, with a JASCO ATR attachment. The PP-0 (original) sample and the further oxidized PP-1 and PP-2 samples as well as the PHAs from each shake flask experiment (designated PHA-PP-x, where x represents the number of prodegraded and oxidized PP sample utilized), were evaluated. Samples were analyzed as they were received in each case.

#### 2.6.3. NMR Analysis

Proton nuclear magnetic resonance (^1^H-NMR) spectra of PHA-PP samples were recorded using a Bruker-Advance spectrometer (Bruker, Rheinstetten, Germany) operating at 600 MHz with Bruker TOPSPIN 2.0 software, using CDCl_3_ as the solvent and tetramethylsilane (TMS) as the internal standard. Spectra were obtained with 64 scans, an 11 μs pulse width, and a 2.66 s acquisition time. The chemical compositions of samples after thermal degradation were calculated from the integration of signals of methyl groups received from 3-HB at 1.28 ppm and 3-HA (HV and HH) at 0.9 ppm.

#### 2.6.4. TGA Characterization

Thermal gravimetric analysis (TGA) was performed with a TGA/DSC1 Mettler-Toledo thermal analyzer at a heating rate of 10 °C min^−1^ in a stream of nitrogen (60 mL/min) from 25 to 600 °C. The obtained TGA data were analyzed with the Mettler-Toledo Star System SW 9.30. The PHA-PP-x samples were evaluated.

#### 2.6.5. PHA Characterization at the Molecular Level by ESI-MS/MS

PHA oligomers for the ESI-MS/MS analyses were obtained via the partial thermal degradation of PHA obtained from shake flask experiments with PP-0, PP-1, and PP-2 samples with TSB, as described above. The partial degradation was performed by controlled moderate-temperature degradation induced by carboxylate in the presence of potassium hydrogen carbonate, KHCO_3_, as described by Johnston et al. [17,28]. The respective amount of polyester and salt, at a ratio PHA/KHCO_3_ equal to 5, was introduced in a vial with ethanol and then the mixture was stirred for 30 min to create a homogeneous suspension that was kept in an oven at 180 °C for 1 h. After partial thermal degradation, the oligomers were purified with Dowex^®^ 50WX4 (hydrogen form; 1:1 wt%). The oligomer was dissolved in chloroform and the mixture was stirred for 4 h. Then, the Dowex^®^ was removed by filtration. The structure at the molecular level of the oligomer samples derived from PHA PP-0 and PHA PP-2 were determined by ESI-MS/MS.

Electrospray ionization mass spectrometry (ESI-MS/MS) analysis was performed using a Thermo Fisher ion trap mass spectrometer (Thermo Fisher Scientific Inc., San Jose, CA, USA). The samples after thermal degradation were dissolved in a water/methanol (1:1; v/v) or chloroform/methanol (1:1) system. The solutions were introduced to the ESI source by continuous infusion using the instrument syringe pump at a rate of 7 µL/min. The ESI source of the LCQ was operated at 4.5 kV and the capillary heater was set to 200 °C. Nitrogen was used as the nebulizing gas. For the ESI-MS/MS experiments, mass-selected mono-isotopic parent ions were isolated and trapped and collisionally activated using helium damping gas, present in the mass analyzer. Each analysis was performed in the positive- ion mode.

## 3. Results

### 3.1. Characterization of PP Carbon Source - Acid Number (AN)

The prodegraded PP-0 sample was titrated and found to have an AN of 14.8, which was compared to the thermally treated samples (Table 1). In all cases, the treated samples had higher AN values and therefore more hydrophilic properties (O–H, C=O groups). By only using ozone, rather than initiators or catalysts, the chance of introducing contaminants was reduced. Using this thermal treatment, the AN of PP-1 to 2 was managed by temperature.

In Table 1, the conditions of the ox-degradation process and titrations with KOH to confirm AN 14.8–84.2, can be seen. To analyze the nature of the oxidized groups in more detail, FTIR was applied. The increase in acid number after oxidation treatments indicated the presence of aldehyde, ketone, or carboxylic acid groups [17,20].

### 3.2. FTIR Analysis of PP Carbon Sources

**Figure 3:**
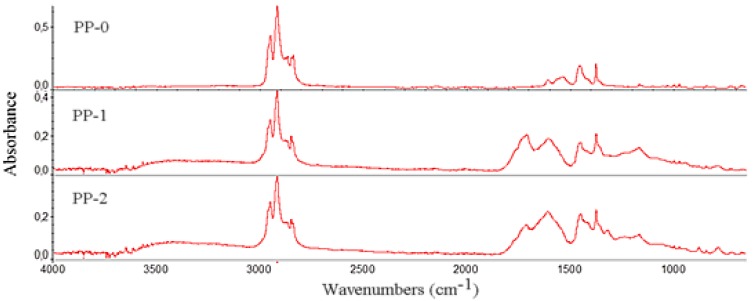
Fourier-transform infrared spectroscopy (FTIR) spectra of samples PP-0 to 2.

The FTIR spectra of the PP-0 sample shown in Figure 3 revealed the following: methyl group (–CH_3_) absorbances can be seen at 2970 cm^−1^ and at 2910 cm^−1^; methylene groups (–CH_2_–) at 2870 cm^−1^; (–CH_2_–) at 2840 cm^−1^; (–CH_2_–) at 1460 cm^−1^; and (–CH_3_) at 1370 cm^−1^. These results closely match the observations of Barbes et al. [29]. A relatively strong absorption band with a maximum around 1720 cm^−1^, corresponding to the stretching vibrations of C=O groups, was observed in the spectrum of the PP-1 and PP-2 samples. The appearance of the carbonyl band in the FTIR spectrum of the PP-1 and PP-2 sample was associated with the presence of carboxylic acid, aldehyde, ketone, or ester groups that arose during the thermal oxidation of the plain PP-0 sample. Additionally, in the PP-1 and PP-2 samples, higher absorption bands, characteristic of the hydroxyl groups, were observed at the region of 1100–1200 cm^−1^ and at 3200–3400 cm^−1^.

Thus, the FTIR spectra show that the increased intensity of OH and C=O groups in the PP-1 and PP-2 samples further increased the hydrophilicity of these samples in comparison to the PP-0 sample. This variation in intensity is expected to make the PP-1 and PP-2 samples more compatible with the organism and more susceptible to microbial attack than the PP-0 sample. The FTIR results also seem to confirm the titration results in Table 2, where PP-1 and PP-2 had higher AN values than PP-0 and therefore were comparatively more hydrophilic carbon sources than the original.

### 3.3. GPC Analysis of the PP Carbon Sources

The oxidation of the PP samples appears to have caused a partial degradation of the polymers, resulting in a general decrease in the weight average molar mass (*M*_w_) (Table 2). The molar mass dispersity index (*DI*) of the samples was calculated by the *M*_w_ and the number average molar mass *(M_n_*) ratio. For the PP-1 sample, there was clearly a reduction of *DI* from PP-0. The PP-2 *DI* value was the lowest, showing the most uniform system.

### 3.4. Bacteria Growth

**Figure 4:**
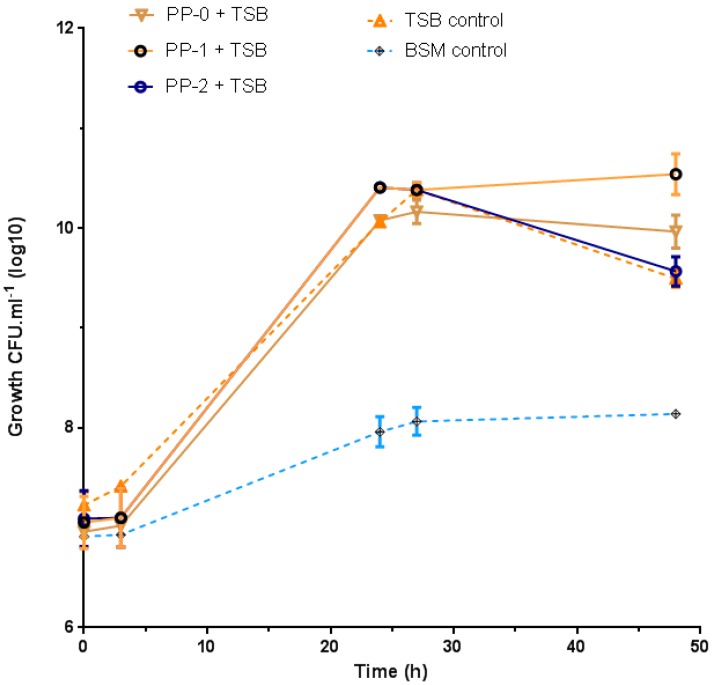
The shake flask experiments of *C. necator* in growth nitrogen-rich tryptone soya broth (TSB) media, with polypropylene (PP) 0 to 2) and basal salts media (BSM) and TSB only controls for comparison. The experiments were conducted over a 48 h period at 150 rpm. Each point is an arithmetic mean of triplicates, while error bars denote the SE of the mean.

The shake flask experiment of *C. necator* in the nitrogen-rich TSB media is shown in Figure 4 over a 48 h period with and without the PP-0 and the pretreated samples PP-1 to 2 along with the TSB and BSM controls. A parallel set of experiments with BSM and PP0 to 2 was also conducted, however, the minimal salts showed very little growth like that shown by the BSM controls, and they produced no PHA. These BSM shake flask results were similar to Johnston et al. [20] where pretreated polystyrene fragments were used as carbon sources, however, in this study, the cell biomass was even less.

The starting CFU numbers of the cultures were between 6.9 and 7.4 log10 CFU/mL. A 2-way multiple ANOVA and Tukey’s multiple comparison showed that there was a significant difference between the control BSM and all of the TSB + PPx as expected (<0.0001).

When the growth was compared to the TSB control, PP-1 had a P-Value of 0.0352, while PP-2 was not significant (P-Value of >0.9999). A possible reason for the PP-1 fragments having the highest growth could be that it also possessed an AN value of 84.2, making it the most hydrophilic additional carbon source. The relative size of the fragments could also provide a platform for some bacterial colonies. PP-0 had the lowest AN, however, it had a relatively strong growth of 3 log10 CFU/mL CFUs, again this could be due to having the largest surface area with PP sheets, rather than PP fragment metabolism as its P-Value was not significant compared to the TSB control. PP-0 also had a similar PHA yield to TSB. The PP-1 shake flask analysis with TSB had the highest yield at 42 %, while PP-2 and PP-0 (under nitrogen-rich conditions) had similar PHA yields to each other in Table 3.

### 3.5. PHA Identification and Characterization

#### 3.5.1. NMR, FTIR, GPC, and Thermal Analysis of PHA

**Figure 5:**
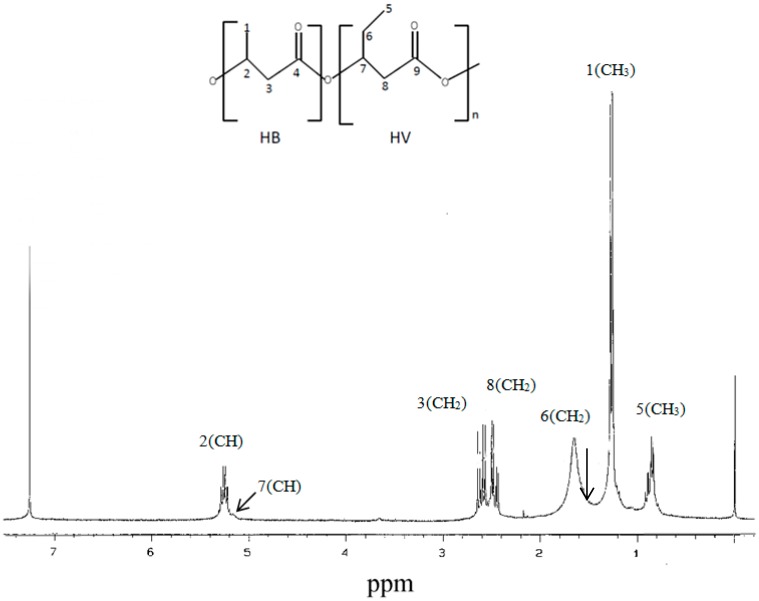
^1^H-NMR of PHAs produced by *C. necator* from TSB with the PP-2 sample as an additional carbon source.

The ^1^H-NMR spectra of PHA-PPx (Figure 5) revealed the presence of characteristic signals corresponding to the protons of methyl groups of 3-hydroxybutyrate (3-HB) and 3-hydroxyalkanoate (3-HA) repeating units at 1.28 ppm and 0.9 ppm, respectively.

The FTIR spectra of the PHA samples produced from the PP carbon source (data not shown) upon comparison to the previous FTIR absorbance spectra of PP (Figure 3) cannot exclude the contamination of the PHA with PP fragments. The characteristic peaks for PP (methyl and methylene groups) in the range of 2840–2970 cm^−1^ as well as the peaks shown over 600–1500 cm^−1^ (which correspond to the C–C aliphatic bonds recorded by Shamala et al. [30]) could be overlapped within the ones of PHA structure.

**Figure 6:**
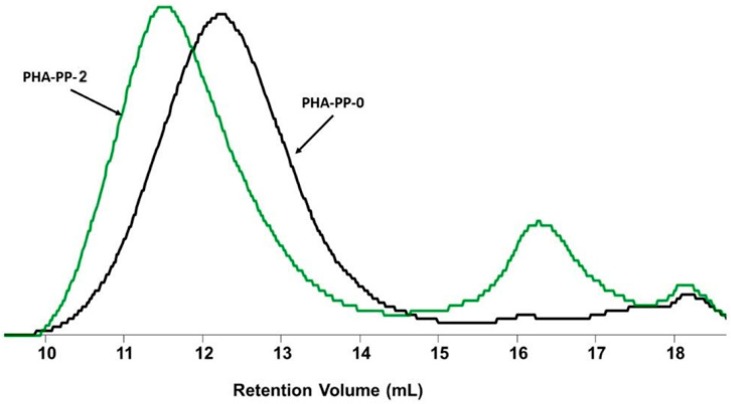
The comparison of the GPC traces of PHA samples produced from PP-0 and PP-2 with TSB by *C. necator* after 48 h.

GPC analysis was used to estimate the molar mass of the obtained biopolymers synthesized by the bacterial shake flask experiments (Table 4). For the sample of PHA-PP-2, the second fraction of lower molar mass was also visible (Figure 6).

The thermal decompositions of PHA produced from the PHA-PP-0 to PP-2 underwent two stages of mass loss. The GPC results suggest that some residue, after the first step of the thermal decomposition of the PHA-PP-2 sample, contained not only PHAs but also PP fragments that survived the post-shake flask process (Table 5) and decomposed at 444.1 °C.

#### 3.5.2. Structural Characterization of PHA with Dual-Stage ESI-MS

The structural characterization (including co-monomer composition and distribution) of biodegradable PHA copolymers was determined with the aid of dual-stage electrospray mass spectrometry (ESI-MS/MS) based on the analysis of the oligomers formed via controlled, partial thermal degradation. Using this method, the uniform PHA oligomers terminated by unsaturated and carboxyl end groups were obtained [5,28,31,32]. The chemical composition of partially degraded PHA copolymers was determined from ^1^H-NMR spectra (data not shown). The obtained results revealed that the content of co-monomeric units in PHA oligomers was the same as that of the original PHA sample.

**Scheme 1:**
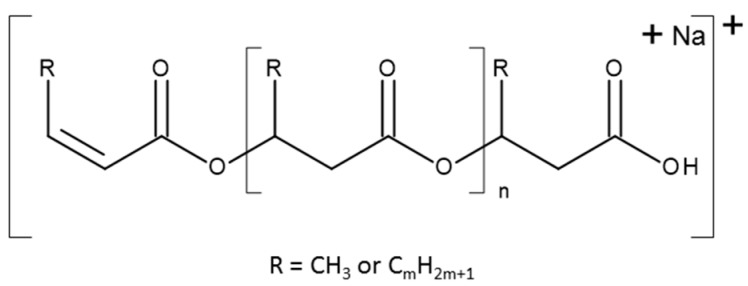
The general chemical structure of the sodium adducts of PHA oligomers presented in the ESI-MS spectrum (Figure 7).

**Figure 7:**
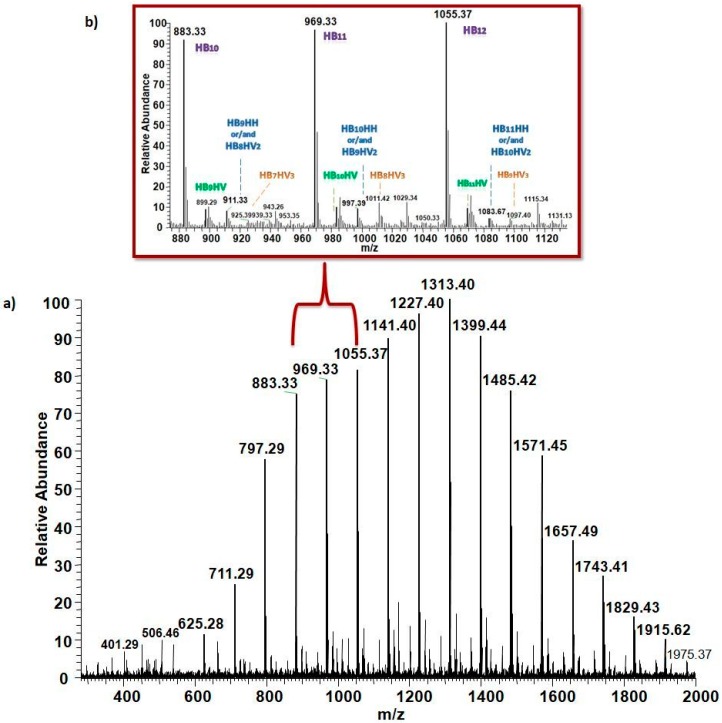
(**a**) The ESI-MS (positive–ion mode) of PHA oligomers formed after partial thermal degradation of sample PHA-PP-0; (**b**) spectral expansion in the mass range at *m/z* 870–1140. The sample was analyzed in a chloroform/methanol (1:1; *v/v*) system.

Figure 7 presents the positive ion ESI-MS spectrum of oligomers formed after partial thermal degradation of sample PHA-PP-0. Due to the high affinity of PHA oligomers toward alkali metals (especially to the sodium), the ESI mass spectrum of these oligomers showed a distribution of singly charged sodium adducts of the individual PHA oligomer chains (composed with HB, HV and/or HH units) terminated by unsaturated and carboxylic end groups with general chemical structures shown in Scheme 1. The signals present in the mass spectrum were grouped in numerous clusters (Figure 7b) due to their different degrees of oligomerization and composition. The differences in the experimental values of *m/z* between the most intensive ions in the neighboring groups of peaks were about 86 Da. This corresponds to the molar mass of the HB unit. The mass difference between the neighboring signals within the clusters was equal to 14 Da, which corresponds to the difference between the molar mass of (differing with the length of side chains) the 3-hydroxyhexanoate (HH, 114 Da), 3-hydroxyvalerate (HV,100 Da), and 3-hydroxybutyrate (HB, 86 Da) co-monomeric units. The signals belonging within the individual clusters, appearing regularly in the mass spectrum, respectively at *m/z* = (n x 86 + 23), *m/z* = ((n-1) x 86 + 100 + 23), and *m/z* = ((n-1) x 86 + 114 +23) or *m/z* = ((n-2) x 86 + 2 x 100 + 23) represent sodium adducts of linear 3HB, 3-HB/3HV or 3-HB/3HH oligomers terminated by unsaturated and carboxylic end groups. The compositions of the ions belonging to the most intense clusters are assigned in Figure 7b.

To verify the structural assignment of the ions present in the ESI-MS spectrum, in particular, to confirm the chemical structure of the end groups as well as the co-monomer sequence distribution (distribution of HA units along the oligomer chains) occurring in the PHA samples synthesized, dual-stage mass spectrometry (ESI-MS/MS) was applied. In this example, the ESI-MS/MS experiments for the ions belonging to the selected 11-mers cluster with nominal masses at *m/z* 969, 983, and 997 were performed. Previously published studies of the fragmentation of individual PHA bio-polyester molecules revealed that the *β*-hydrogen rearrangement was the main mechanism inducing fragmentation of such polyesters via ester bond cleavage [5,6,31,32]. In the *β*-hydrogen transfer, the rearranged hydrogen is attached to the carbonyl oxygen, and this leads to cleavage of the alkyl-oxygen bond, resulting in the formation of two ions: one bearing a carboxylic acid end group and the other terminating with a crotonate group [33].

**Figure 8:**
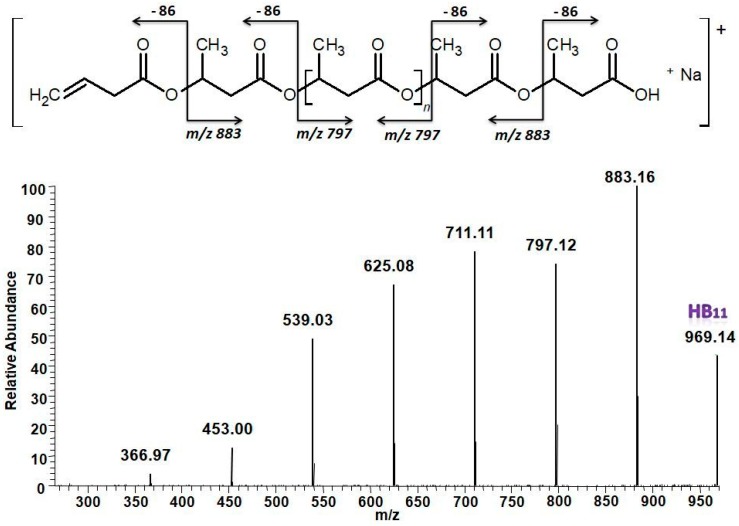
The ESI-MS/MS fragmentation spectrum of selected sodium adduct of [HB_11_ + Na]^+^ oligomers, at *m/z* 969 together with the theoretical fragmentation pattern.

The ESI-MS/MS product ions spectrum for the selected sodium adduct of oligomer [HB_11_ + Na ]^+^ at *m/z* 969 is presented in Figure 8.

The fragmentation of this ion, which resulted from the random breakage of ester bonds along the oligomer chain, led to the formation of only one set of oligo(3-hydroxybutyrate) product ions occurring at the values *m/z* equal to 883, 797, 711, 625, 539, 453, and 367, terminated by crotonate and carboxyl end groups. For example, the first ion from this series at *m/z* 883 can be produced by the cleavage of the ester bond and the loss of a neutral molecule of crotonic acid (86 Da), which can take place from both ends of the parent ion *m/z* 969. The comparison of experimental ESI-MS/MS spectra with theoretical fragmentation patterns of this ion confirmed that the most intensive ion in this cluster corresponded to sodiated linear 3-hydroxybutyrate homopolymer chains built with the 11 HB repeat units successively connected by ester bonds.

**Figure 9:**
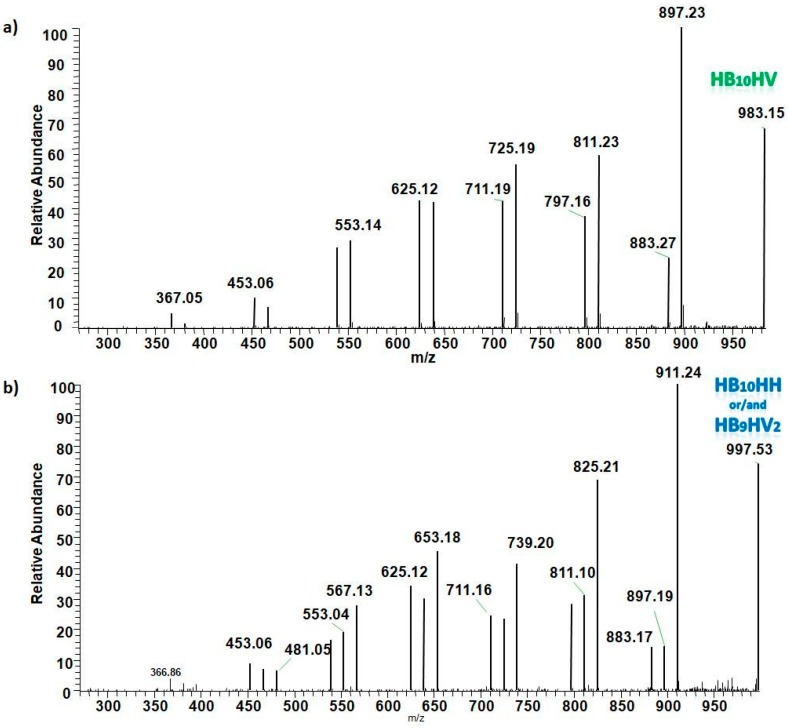
The ESI-MS/MS fragmentation spectra of selected sodium adducts of PHAs of [HB_10_HV + Na]^+^ oligomers at *m/z* 983 (**a**) and of HB_9_HV_2_+ Na]^+^ or/and [HB_10_HH + Na]^+^ oligomers at *m/z* 997 (**b**).

In Figure 9a,b, the ESI-MS/MS spectra of the selected parent sodium adducts of PHA oligomers at *m/z* 983 and at *m/z* 997 are shown.

Due to the random distribution of HB, HV, and HH units along the PHA co-oligomer chains, which yields the highest number of possible sequences in those oligomers, the spectra presented in Figure 9 are more complex than for the homopolymer presented in Figure 8. The fragment ions present in the MS/MS spectra (Figure 9a,b), which are formed via the random breakage of ester bonds along the oligomer chain, are grouped in clusters containing two fragment ions (in the case of [HB_10_HV + Na]^+^ or three fragment ions (in the case of a mixture of [HB_10_HH + Na]^+^ and/or [HB_9_HV_2_ + Na]^+^). The individual clusters containing fragment ions with the same degree of oligomerization, but differing in chemical composition (different content of HB and HV units, or HB and HH units).

**Scheme 2:**
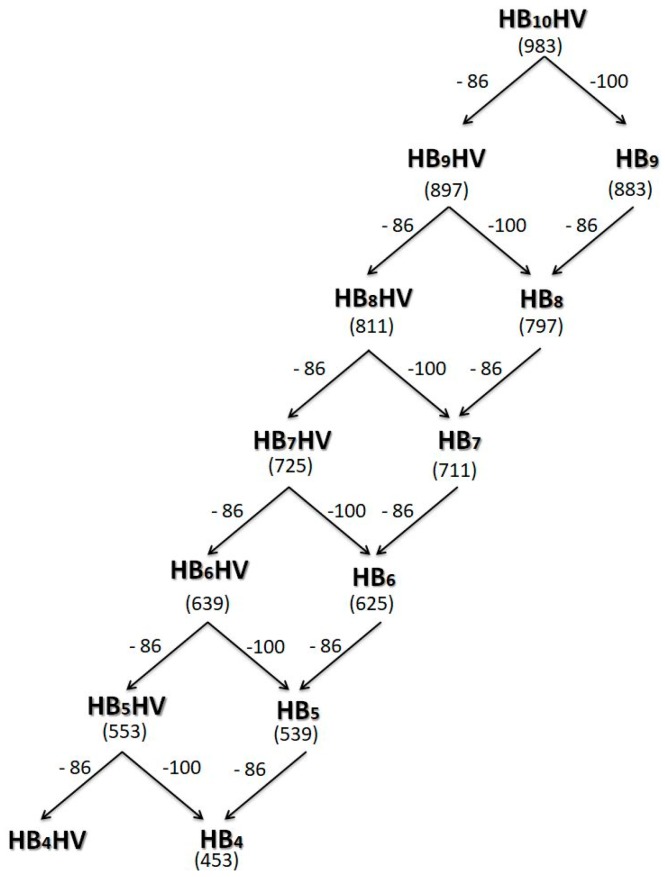
The theoretical fragmentation patterns of the selected sodiated [HB_10_HV + Na]^+^ oligomers at *m/z* 983 Figure 9a.

**Scheme 3:**
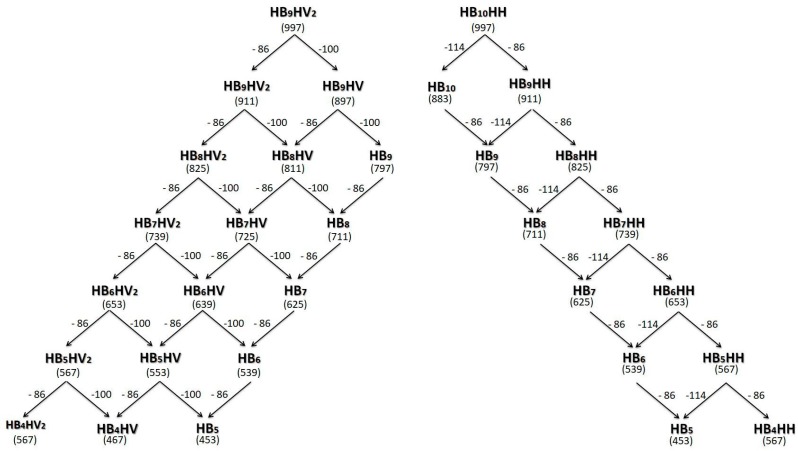
The theoretical fragmentation patterns of the selected sodium adduct [HB_9_HV_2_ + Na]^+^ or/and [HB_10_HH + Na]^+^ oligomers at *m/z* 997 (Figure 9b).

In the case of sodium adduct of [HB_10_HV + Na]^+^ oligomers, at *m/z* = 983 the first cluster with two fragment anions at *m/z* 897 and *m/z* 883 can be produced by the cleavage of ester bonds and the losses of crotonic acid (86 Da) or 2-pentenoic acid (100 Da), respectively (Figure 9a). While in the case of the sodium adduct of [HB_10_HH + Na]^+^ or/and [HB_9_ HV_2_ + Na]^+^ oligomers, at *m/z* = 997 the first clusters with three fragment anions at *m/z* 911, *m/z* 897, and *m/z* 883 are produced by the cleavage of ester bonds and the losses of crotonic acid (86 Da), 2-pentenoic acid (100 Da), or 2-hexenoic acid (114), respectively (Figure 9b). The formation of the fragment ion with nominal mass *m/z* 883 clearly demonstrates the presence of the HH unit in the oligomer studied [5]. The theoretical fragmentation patterns of the selected sodiated [HB_10_HV + Na]^+^ oligomers, at *m/z* 983 and [HB_9_HV_2_ + Na]^+^ or [HB_10_HH + Na]^+^ oligomers at *m/z* 997 are presented in Scheme 2 and Scheme 3, respectively.

The experimental mass spectra presented in Figure 8 and Figure 9 confirmed that the resulting PHA samples were random copolymers that contained both the 3-HB homo-polyester molecules as well as 3-HB/HV and 3-HB/HH co-polyester molecules.

**Figure 10:**
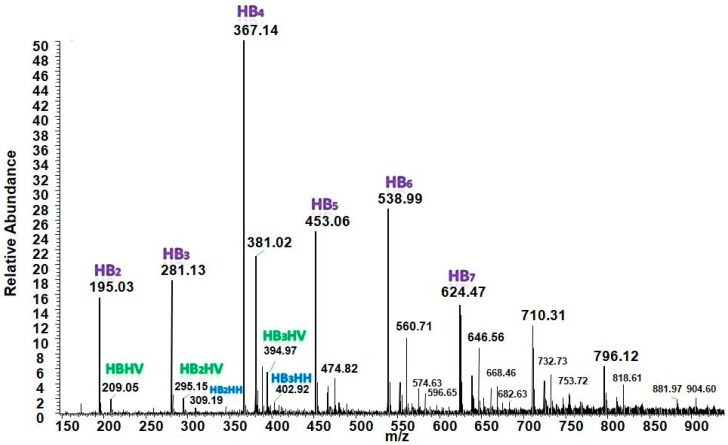
ESI-MS (positive–ion mode) of the water-soluble fraction of PHA oligomers formed after the partial thermal degradation of the PHA-PP2 sample.

In order to verify the molecular structure of PHA derived from thermally treated prodegraded PP, the sample obtained via partial thermal degradation of PHA PP-2 was analyzed. The oligomer sample obtained contained the fraction which was dissolved in water. For structural characterization of this fraction, dual-stage electrospray mass spectrometry (ESI-MS/MS) was also applied and the acquired spectrum is presented in Figure 10.

**Figure 11:**
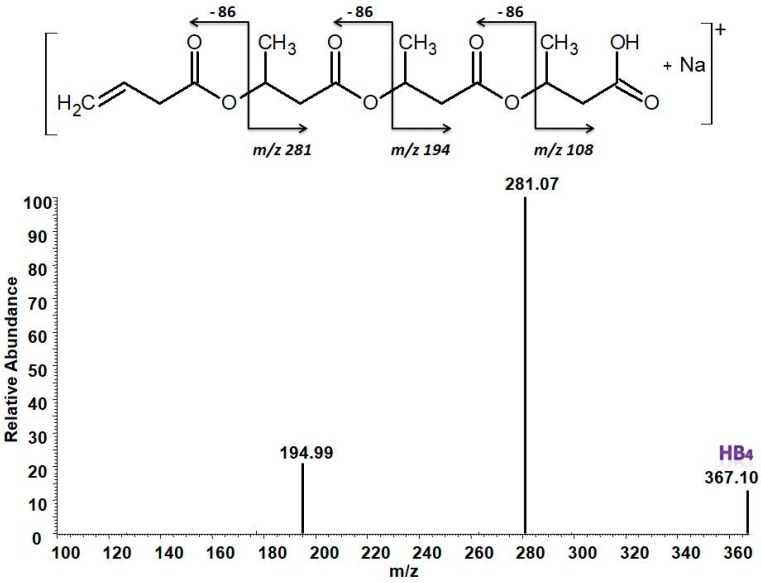

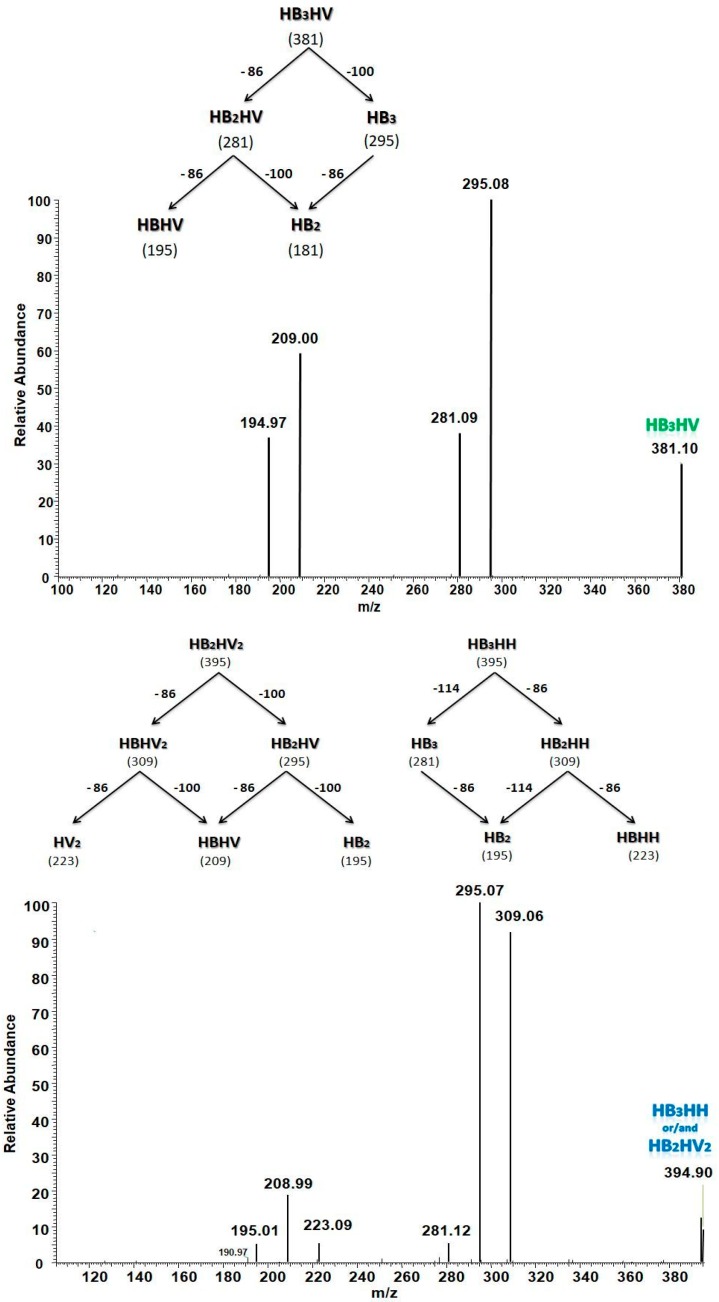
The ESI-MS/MS fragmentation spectra of the selected parent sodium adducts of [HB_4_ + Na]^+^ at *m/z* 367, [HB_3_HV + Na]^+^ at *m/z* 381, and [HB_2_HV_2_ + Na]^+^ or/and [HB_3_HH + Na]^+^ at *m/z* 395 oligomers selected from the ESI-mass spectrum in Figure 10.

The ESI-mass spectrum obtained for a water soluble fraction of the PHA oligomers is similar to that presented in Figure 7. The visible differences were connected with the mass range. The ESI-MS/MS spectra of the selected parent sodium adducts [HB_4_ + Na]^+^ at *m/z* 367, [HB_3_HV + Na]^+^ at *m/z* 381 and of the sodium adducts [HB_2_HV_2_ + Na]^+^ or [HB_3_HH + Na]^+^ oligomers at *m/z* 395 are present in Figure 11.

The results of ESI-MS/MS fragmentation experiment performed for the selected ions, which represent the individual PHA oligoester chains, confirmed that the water-soluble fraction of PHA oligomers is also composed of both the 3-HB oligomers as well as 3-HB/HV and 3-HB/HH co-oligoester oligomers. The formation of the fragment ion with nominal mass at *m/z* 281 clearly demonstrates the presence of a HH unit in the oligomer studied.

## 4. Discussion

The originally reported utilization of pro-degraded PP as an additive carbon supplement in the bacterial production of PHA would give PP an extended usefulness life-span, as opposed to it adding to the current high levels of waste plastic pollution found around the globe, as discussed in the introduction. From the Table 1 results, PP-1 received the highest AN value in the thermal pretreatment with ozone at 60 °C for 20 h. This process does appear to have added ketone and aldehyde groups to the relatively saturated waste PP, making it a more facile carbon source for the shake flask procedure as seen in the growth curve (Figure 4). Although some of the other PP oxidized samples may have had more intense readings for ketone and aldehyde functional groups, the prolonged heat treatment is likely to have caused cross-linking structural changes that hindered bacterial uptake. The GPC data showed a decrease in the average molar mass of the PP specimens from PP-0 to PP-2, with PP-1 and PP-2 being equal at 2000 and seemingly being a good structural compromise for bacterial uptake.

As expected from the shake flask data, there was no production of PHAs when the minimal salt media and PP specimens were used as the sole carbon source. Again, from the FTIR and GPC results, the molecular size and hydrophilicity (AN = 84.2) aids the absorption of PP-1 by bacteria into the *β*-oxidation pathway for PHA production, supporting the notion that it was the best carbon supplement developed in this study. From Figure 4 and Table 5, TSB and the addition of PP-0 to 2 did not have a negative effect on the growth of *C. necator* at 30 °C. Some previous experiments have shown that plastics like PE can have adverse growth effects on Gram-positive and Gram-negative bacteria [34,35]. The antimicrobial action of some plastics could be due to the presence of antimicrobial moieties such as fluorine or chlorine caught within the chains of the polymers. These elements could later be discharged in the midst of the thermal treatment process where they could then be released into the ambient growth media, altering overall cell proliferation [36]. PP-1 had the highest yield of PHA at 42 %, which was 20 % higher than the TSB control results.

Proton NMR analysis was exploited in the assessment of the average structure across a whole sample of the copolymers synthesized for each PP carbon source. The ^1^H-NMR spectra of PHA-PPx showed the expected signals corresponding to the protons of 3-hydroxybutyrate (3-HB) and 3-hydroxyvalerate (3-HV) repeating units, which differed considerably from PHAs produced from TSB only. However, ESI-MS/MS was needed to determine the structure of the copolymers at the molecular level. This approach was included in the structural studies of PHAs produced from the pro-degraded PP, since a method is known to provide more details than ^1^H-NMR alone as it can corroborate whether copolymers are a co-block polymer or arbitrarily distributed [26]. The series of the cluster ions in the mass spectrum were sodium adducts derived from PHA with HB, HV, and HH chain units terminated by unsaturated and carboxylate groups. To further verify the PHA structure, the PHA-PP-0 and PHA-PP-2 samples were selected for dual MS studies and after their partial degradation, it was found that clusters with HB, HV, and HH repeat units with randomly distributed HV and HH units along the oligoester chains were formed.

The significance of the biopolymer structures of PHA obtained is that properties such as melting point or mechanical strength are changed, meaning that the applications of these bioplastics are different than that of the PHB homopolymer. PHAs are known to be marine biodegradable polymers and are easily broken-down by bacteria and fungi. Use of PHA for netting, packaging, or as an additive in 3D printing filaments creates opportunities for other uses in diverse marine applications, where biodegradation process gives an added value [37]. One of the additional reasons that PHA is such a good candidate for marine use, as opposed to traditional petrol-based plastics or PLA, is its rate of biodegradation in the ambient environment, because PLA, for example, needs a significant reduction in molar mass *via* hydrolysis before biodegradation can start [37].

## 5. Conclusions

To the best of our knowledge, we have originally reported the viability of using waste PP as an additional carbon source for the production of PHA, using an integrated approach that included bacterial shake flask procedures, FTIR, GPC, TGA, NMR, and ESI-MS/MS. In summary, the conditions needed to form viable PP as an additional carbon source were found. The PP-1 and PP-2 samples were found to be the most economically viable and treated PPx samples functioned as an additional carbon source to produce PHA copolymers that contained randomly distributed HV and HH repeat units along their PHA chains, as revealed by electrospray dual-stage mass spectrometry at the molecular level.

## 6. Patents

Partial degradation of PHA is a subject of an EU patent entitled: Process for Controlled Degradation of Polyhydroxyalkanoates and Products Obtainable there from. EP 2 346 922 B1. International publication number: WO 2010/044112 (22.04.2010 Gazette 2010/16). Date of filing: 15.10.2008. Date of publication of application: 27.07.2011.

## Figures and Tables

**Table 1:** Acid number (AN) and conditions used for the thermal degradation of PP-0 to 2.

Sample	AN [mg KOH/g]	Process Conditions
**PP-0**	14.8	N/A
**PP-1**	84.2	80 °C, O_3_/O_2_, 20 h
**PP-2**	66.3	100 °C, O_3_/O_2_, 20 h

**Table 2:** The weight average molar mass (*M*_w_), number average molar mass (*M*_n_) and dispersity index (*DI*) of the PP-0 to 2 samples used in this study.

Sample	*M_w_*	*M_n_*	*DI* (*M*_w_/*M_n_*)
PP-0	148,000	18,000	8.2
PP-1	12,000	2200	5.2
PP-2	4000	1600	2.6

**Table 3:** The percentage yield of PHA synthesized by *C. necator* using 2 g/L PP conducted over a 48 h period at 30 °C (150 rpm). ND = non-detected; CDW = cellular dry weight.

Media	Average CDW (g/L)	Average PHA (g/L)	PHA (%*w/w*)
BSM only	0.09 ± 0.10	ND	ND
TSB only	2.18 ± 0.20	0.48 ± 0.06	22%
PP-0 + BSM	0.07 ± 0.03	ND	ND
PP-0 + TSB	2.26 ± 0.06	0.58 ± 0.02	26%
PP-1 + TSB	3.22 ± 0.10	1.36 ± 0.20	42%
PP-2 + TSB	2.82 ± 0.10	0.78 ± 0.02	28%

**Table 4:** The GPC results of the PHA-PPx samples produced after 48 h in shake flask analysis with PP-0 to 2 as additional carbon sources.

Sample Name	M_w_	M_n_	*DI*
PHA-PP-0	410,000	139,000	2.9
PHA-PP-1	676,000	221,000	3.0
PHA-PP-2	710,000	259,000	2.7

**Table 5:** Thermogravimetric parameters for PHA samples produced after 48 h shake flask procedures with PP samples utilized as additional carbon sources in TSB media.

Sample name	Step 1		Step 2	
	**T_max_ (°C)**	**Weight loss (%)**	**T_max_ (°C)**	**Weight loss (%)**
PHA-PP-0	291.4	86.6	-	97.3
PHA-PP-1	294.7	88.3	-	98.7
PHA-PP-2	294.2	71.6	444.1	97.7
